# Investigating potential immune mechanisms of trilaciclib administered prior to chemotherapy in patients with metastatic triple-negative breast cancer

**DOI:** 10.1007/s10549-023-07009-8

**Published:** 2023-07-07

**Authors:** Antoinette R. Tan, Joyce O’Shaughnessy, Subing Cao, Sarah Ahn, John S. Yi

**Affiliations:** 1 Levine Cancer Institute, Atrium Health, 1021 Morehead Medical Drive, Charlotte, NC 28204 USA; 2 Baylor University Medical Center, Texas Oncology Dallas, US Oncology Research, 3410 Worth Street, Suite 400, Dallas, TX 75246 USA; 3 G1 Therapeutics, Inc., 700 Park Offices Drive, Suite 200, Research Triangle Park, NC 27709 USA

**Keywords:** CDK4/6, Chemotherapy, Immune profiling, Metastatic triple-negative breast cancer, Trilaciclib

## Abstract

**Purpose:**

In a phase II trial in patients with metastatic triple-negative breast cancer (mTNBC; NCT02978716), administering trilaciclib prior to gemcitabine plus carboplatin (GCb) enhanced T-cell activation and improved overall survival versus GCb alone. The survival benefit was more pronounced in patients with higher immune-related gene expression. We assessed immune cell subsets and used molecular profiling to further elucidate effects on antitumor immunity.

**Methods:**

Patients with mTNBC and ≤ 2 prior chemotherapy regimens for locally recurrent TNBC or mTNBC were randomized 1:1:1 to GCb on days 1 and 8, trilaciclib prior to GCb on days 1 and 8, or trilaciclib alone on days 1 and 8, and prior to GCb on days 2 and 9. Gene expression, immune cell populations, and Tumor Inflammation Signature (TIS) scores were assessed in baseline tumor samples, with flow cytometric analysis and intracellular and surface cytokine staining used to assess immune cell populations and function.

**Results:**

After two cycles, the trilaciclib plus GCb group (*n* = 68) had fewer total T cells and significantly fewer CD8+ T cells and myeloid-derived suppressor cells compared with baseline, with enhanced T-cell effector function versus GCb alone. No significant differences were observed in patients who received GCb alone (*n* = 34). Of 58 patients in the trilaciclib plus GCb group with antitumor response data, 27 had an objective response. RNA sequencing revealed a trend toward higher baseline TIS scores among responders versus non‑responders.

**Conclusion:**

The results suggest that administering trilaciclib prior to GCb may modulate the composition and response of immune cell subsets to TNBC.

## Introduction

Triple-negative breast cancer (TNBC) is a specific subtype of breast cancer that is associated with high invasiveness, high metastatic potential, proneness to relapse, and poor prognosis. Patients with TNBC have fewer treatment options available to them than those with other types of invasive breast cancer [1–4]. Cytotoxic chemotherapy is the primary treatment option for patients with programmed death-ligand 1 (PD-L1)-negative disease [4, 5], whereas the combination of chemotherapy plus pembrolizumab is the preferred first-line treatment option for patients with PD-L1-positive tumors (the overall rate of PD-L1 positivity in TNBC ranges from 20 to 60%, depending on the assays and methods used) [6–9]. Other treatment options include poly(adenosine diphosphate-ribose) polymerase inhibitors for patients with *BRCA*-mutated tumors (reported prevalence rates varying from 10 to 20%) [10, 11], and sacituzumab govitecan, a Trop-2-directed antibody–drug conjugate, for the treatment of patients with unresectable locally advanced or metastatic TNBC (mTNBC) who have received two or more prior lines of systemic therapy [12]. Despite the emergence of these new therapies, many patients with locally advanced TNBC or mTNBC have no options other than standard chemotherapy, which is commonly associated with toxicities that can adversely impact quality of life [13].

Trilaciclib is an intravenous myeloprotection therapy that is administered as a 30-min infusion within 4 h prior to the start of chemotherapy on each day chemotherapy is administered. Trilaciclib transiently arrests cyclin-dependent kinase 4/6 (CDK4/6)-dependent hematopoietic stem and progenitor and immune cells in the G1 phase of the cell cycle during chemotherapy exposure, protecting these cells from chemotherapy-induced damage [14–16]. In 2019, trilaciclib received breakthrough designation from the US Food and Drug Administration (FDA) and, in 2021, was approved by the FDA to decrease the incidence of chemotherapy-induced myelosuppression in adult patients when administered prior to a platinum/etoposide-containing regimen or topotecan-containing regimen for extensive-stage small cell lung cancer (ES-SCLC) on the basis of the results from three randomized, placebo-controlled phase II studies [17–19].

Trilaciclib has been shown to favorably alter the tumor immune microenvironment in in vivo murine syngeneic models [15, 20, 21], in an ex vivo patient-derived organotypic tumor spheroid culture system [20], and in a clinical setting in patients with ES-SCLC [15, 17]. Specifically, trilaciclib has been shown to enhance T-cell activation and the production of cytokines and chemokines [15, 20], to promote a favorable tumor immune microenvironment by increasing the intratumoral ratio of effector T cells to regulatory T cells (Tregs) and the number of activated T cells in the periphery [15], to inhibit immunosuppression by Tregs [15, 20], to significantly increase the expansion of T-cell clones [15, 17], and to enhance the induction of memory cluster of differentiation (CD)8+ T cells [21]. Consistent with its known mechanism of action, trilaciclib may elicit some of these immune effects by protecting lymphocytes from chemotherapy-induced damage [22].

The efficacy and safety of trilaciclib in patients with mTNBC have been investigated in a randomized phase II trial (NCT02978716) [23, 24]. Treatment with trilaciclib prior to gemcitabine plus carboplatin (GCb) did not lead to a significant improvement in duration and occurrence of severe neutropenia (primary endpoint); however, overall survival (OS; secondary endpoint) was improved for patients who received trilaciclib plus GCb compared with those who received GCb alone (median 19.8 vs. 12.6 months, respectively) [23]. In subgroup analyses, OS was prolonged irrespective of CDK4/6 dependence and PD-L1 status, but benefit was greater in the PD-L1-positive population. OS was also more pronounced in, but not exclusive to, patients with higher immune-related gene expression [24]. Lastly, administering trilaciclib enhanced T-cell activation, as evidenced by an enrichment of new T-cell clones and decreased Simpson clonality in peripheral blood [23, 24].

The aim of the current analysis was to further investigate potential immune mechanisms of trilaciclib in mTNBC through the analysis of immune cell subsets and molecular profiling in peripheral blood and tumor samples, respectively.

## Materials and methods

### Study design and participants

This analysis is based on data from a multicenter, randomized, open-label, phase II trial including patients aged ≥ 18 years with mTNBC who had received up to two prior chemotherapy regimens for locally recurrent TNBC or mTNBC (NCT02978716) [23, 24]. Patients were randomized (1:1:1) to 21-day treatment cycles: GCb (gemcitabine 1000 mg/m^2^, carboplatin area under the curve 2) alone on days 1 and 8; trilaciclib 240 mg/m^2^ within 4 h prior to GCb on days 1 and 8; or trilaciclib alone on days 1 and 8 and trilaciclib within 4 h prior to GCb on days 2 and 9.

### Antitumor efficacy endpoints and assessments

Efficacy and survival outcomes were analyzed as prespecified secondary endpoints and included objective response rate (confirmed complete or partial response) assessed in response-evaluable patients, and progression-free survival and OS, assessed in the intention-to-treat population. Objective response rate and progression-free survival were investigator assessed according to Response Evaluation Criteria in Solid Tumours (RECIST) version 1.1, based on the May 15, 2020, data cut-off. For tumor assessment, computed tomography or magnetic resonance imaging was performed at screening and at protocol-specified intervals (every 9 weeks for the first 6 months, then every 12 weeks thereafter) until disease progression, withdrawal of consent, or receipt of subsequent anticancer therapy. OS was analyzed following the final database lock on July 17, 2020. Genetic and/or expression markers in blood and tumors and immunologic markers, including PD-L1 expression, were analyzed as post hoc exploratory objectives. Baseline PD-L1 status was measured using the Ventana SP142 PD-L1 assay; tumors were scored as PD-L1 positive if the proportion of PD-L1-expressing tumor-infiltrating immune cells was ≥ 1% and PD-L1 negative if < 1%, per the assay interpretation guide for TNBC tumors [25].

### Peripheral immune cell population and function analysis

Peripheral blood was collected prior to and during treatment for flow cytometric analysis; for the purposes of this analysis, samples were collected prior to treatment on the first day of the first and third treatment cycles (C1D1 and C3D1, respectively). Blood was collected in Cyto-Chex^®^ (Streck) and sodium heparin tubes and shipped at ambient temperature on the day of collection for processing. Whole blood was used for intracellular cytokine staining and surface staining. Staining and flow cytometric assays were performed by a contract research organization (Covance Central Laboratory Services; Indianapolis, Indiana, USA).

### Tumor gene expression analysis

Genomic DNA and total RNA were simultaneously purified and sequenced as previously described from formalin-fixed, paraffin-embedded (FFPE) diagnostic tumor samples collected at baseline [23]. Purification was performed using the AllPrep DNA/RNA FFPE kit (QIAGEN; Germantown, Maryland, USA). Libraries were prepared using TruSeq RNA and DNA Exome kits for RNA-Seq and DNA-Seq, respectively (Illumina; San Diego, California, USA). Cluster generation and sequencing of libraries were performed on the Illumina HiSeq system. Gene expression read counts and fragments per kilobase of exon per million mapped reads (FPKM) were quantified using RNA-Seq by Expectation Maximization (RSEM) software [26]. RNA-Seq samples in which < 30% of RNA fragments were > 200 nucleotides in length (DV_200_) were excluded from the analysis. Differentially expressed genes between trilaciclib responders (complete or partial response) and non‑responders (stable or progressive disease), at an adjusted *P* value of < 0.05, were identified using the DESeq2 package [27]. Gene set enrichment analysis (GSEA) was performed using GSEA_4.1.0 software (number of permutations: 10,000; permutation type: phenotype) [28, 29] using the Kyoto Encyclopedia of Genes and Genomes (KEGG) database (c2.cp.kegg.v7.4).

### Tumor immune microenvironment analysis

The Tumor Inflammation Signature (TIS) [30] was used to assess the tumor immune microenvironment at baseline. The TIS is an investigational 18-gene signature that detects a pre-existing but suppressed adaptive immune response within tumors by measuring the expression of genes associated with antigen-presentation cell abundance (*PSMB10, HLA-DQA1*, *HLA-DRB1, CMKLR1*), T-cell and natural killer (NK)-cell abundance (*HLA-E*, *NKG7*, *CD8A*), interferon (IFN) activity (*CCL5*, *CXCL9*, *CD27*, *CXCR6*, *IDO1*, *STAT1*), and T-cell exhaustion (*TIGIT*, *LAG3*, *CD274*, *PDCDILG2, CD276*) [30]. TIS or signature scores were calculated as an average of the expression values (quantile-normalized and log_10_-transformed) of the respective gene sets.

### Statistical methods

Statistical comparisons of cell numbers/ratios and TIS scores for different time points and patient groups were performed using the Wilcoxon signed-rank test. Plots were created using the ggplot2 and EnhancedVolcano R packages [31, 32].

## Results

### Participants and treatment

As of July 17, 2020, median (range) duration of follow-up was 8.4 (0.1–25.7) months for the 34 patients who received GCb alone, 14.0 (1.3–33.6) months for the 33 patients who received trilaciclib prior to GCb on days 1 and 8, and 15.3 (3.5–33.7) months for the 35 patients who received trilaciclib alone on days 1 and 8 and trilaciclib prior to GCb on days 2 and 9. Antitumor response status was available for 58 of the 68 patients who received trilaciclib prior to GCb: 27 patients (46.6%) had an antitumor response with trilaciclib plus GCb (trilaciclib responders), and 31 (53.4%) had no response (non‑responders).

### Analysis of immune subsets and T-cell function at C1D1 versus C3D1 in patients receiving trilaciclib prior to GCb or GCb alone

**Fig. 1:**
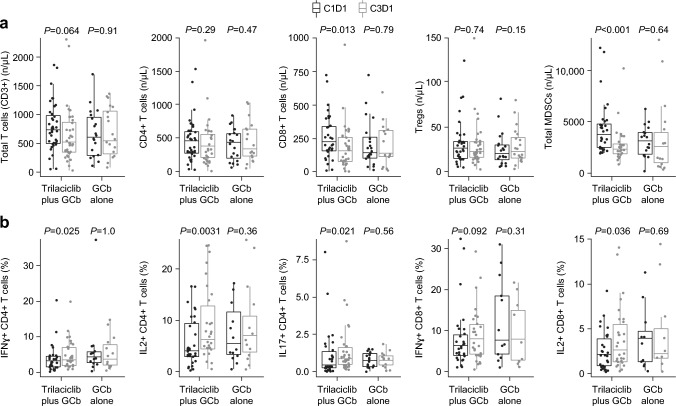
Changes to **a** immune cell populations and **b** T-cell function in peripheral blood over two cycles (C1D1 vs. C3D1) in patients receiving trilaciclib prior to GCb or GCb alone. *C1D1* cycle 1, day 1 *C3D1* cycle 3, day 1 *CD* cluster of differentiation, *GCb* gemcitabine plus carboplatin, *IFNγ* interferon gamma, *IL* interleukin, *MDSC* myeloid-derived suppressor cell, *Treg* regulatory T cell

Patients who received trilaciclib prior to GCb had fewer total T cells (*P* = 0.064) and significantly fewer CD8+ T cells (*P* = 0.013) and myeloid-derived suppressor cells (MDSCs; *P* < 0.0001) at C3D1 compared with C1D1, whereas no significant differences were observed between these time points in patients who received GCb alone (Fig. 1a). Administering trilaciclib prior to GCb greatly enhanced T-cell effector function compared with administering GCb alone, as evidenced by significant increases in the number of cytokine-producing CD4+ and CD8+ T cells (Fig. 1b) from C1D1 to C3D1. No significant differences from baseline in T-cell effector functions were observed in patients who received GCb alone.

### Immune cell populations and T-cell function analysis among trilaciclib responders versus non-responders

**Fig. 2:**
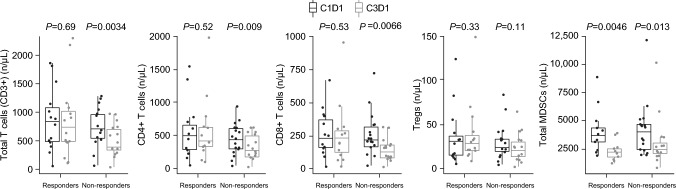
Changes to immune cell populations in peripheral blood over two cycles (C1D1 vs. C3D1) for trilaciclib responders and non-responders. *C1D1* cycle 1, day 1, *C3D1* cycle 3, day 1, *CD* cluster of differentiation, *MDSC* myeloid-derived suppressor cell, *Treg* regulatory T cell

**Fig. 3:**
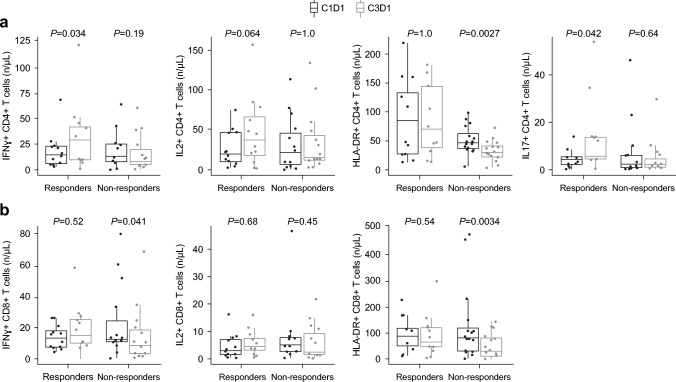
Changes to **a** CD4+ and **b** CD8+ T-cell function in peripheral blood over two cycles (C1D1 vs. C3D1) for trilaciclib responders and non-responders. *C1D1* cycle 1, day 1, *C3D1* cycle 3, day 1, *CD* cluster of differentiation, *HLA-DR* human leukocyte antigen—DR isotype, *IFNγ* interferon gamma, *IL* interleukin

To determine if the impact on T-cell effector function was attributable to clinical outcomes, data from responders and non‑responders who were treated with trilaciclib were compared. After two cycles, T-cell numbers were maintained in trilaciclib responders but significantly reduced in non‑responders (*P* = 0.0034; Fig. 2), with significant reductions in CD4+ T cells (*P* = 0.009) and CD8+ T cells (*P* = 0.0066) contributing to the overall reduction. Tregs were maintained among both responders and non‑responders, whereas MDSCs were significantly reduced among both responders (*P* = 0.0046) and non‑responders (*P* = 0.013; Fig. 2). T-cell function was maintained or improved in responders but was maintained or reduced in non‑responders. Human leukocyte antigen–DR isotype (HLA-DR) expression, a marker of T-cell activation, was also downregulated in trilaciclib non‑responders (Fig. 3).

### Tumor gene expression analysis (trilaciclib responders vs. non-responders at baseline)

**Fig. 4:**
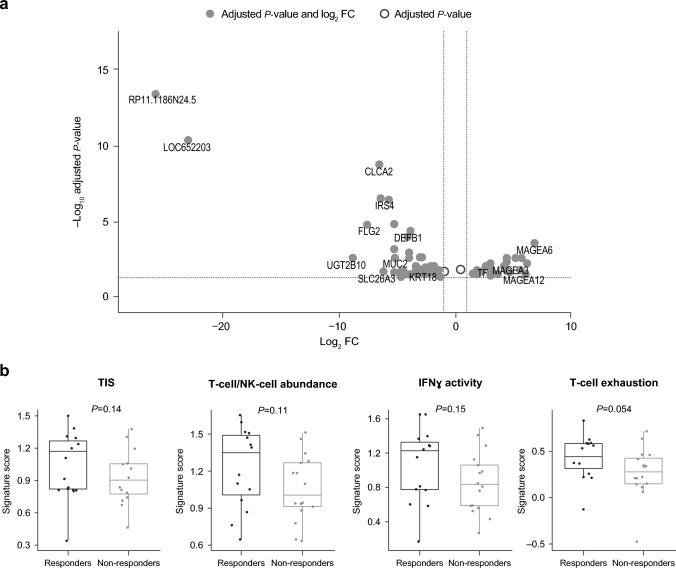
**a** Differential gene expression analysis (adjusted *P* value < 0.05; red dots, │log_2_FC│ > 1; blue dots, │log_2_FC│ ≤ 1) and **b** tumor inflammation signatures in tumor samples from trilaciclib responders and non-responders. False discovery rate < 0.25. *FC* fold change, *IFNγ* interferon gamma, *NK* natural killer, *TIS* tumor inflammation score

Analysis of tumor samples revealed 69 differentially expressed genes (adjusted *P* < 0*.*05) between trilaciclib responders (*n* = 15) and non‑responders (*n* = 17) at baseline (Fig. 4a). In total, 23 genes were upregulated and 46 genes were downregulated (Table 1).

**Table 1:** Upregulated and downregulated genes for trilaciclib responders versus non-responders at baseline

Upregulated genes	Downregulated genes
*MAGEA3, MAGEA6, MAGEA12, CSAG1, OR8G1, PAGE2, BC011773, GABRA3, CSAG3, TMEM213, POTEG, MEF2B, ALOX12B, KRT81, BNC1, TF, BHLHA15, GTSF1, TUBB1, C4orf26, SPAG4, DIS3L, WHAMM*	*TMEM237, TTLL7, KRT18, CRAT, CMBL, SNORA65, SLC7A2, ENPP3, SPTB, FGFR4, ABCC6, SOX11, AR, REEP6, RBM20, NEURL, ZNF729, TMEM45B, MAPT, TDRD1, CYP4Z1, AGR2, APOB, DEFB1, FAM5C, MUC2, ABCC11, ALB, TFAP2B, DLK1, FGB, FGG, CRISP3, ZFP42, FLG, CDSN, UGT2B11, DIO1, MUCL1, SLC26A3, IRS4, CLCA2, FLG2, UGT2B10, LOC652203, RP11-1186N24.5*

KEGG pathways upregulated in trilaciclib responders (FDR < 0.25) were T-cell receptor signaling, antigen processing and presentation, NK-cell–mediated cytotoxicity, nucleotide-binding oligomerization domain (NOD)-like receptor signaling, Toll-like receptor signaling, cytosolic DNA sensing, graft-versus-host disease, and glycosphingolipid biosynthesis. Analysis of immune gene signatures revealed trends toward a higher overall TIS score at baseline among responders versus non‑responders (Fig. 4b). Trends toward increased TIS score were observed for T-cell/NK-cell abundance, IFN activity, and T-cell exhaustion in trilaciclib responders.

## Discussion

Data from this exploratory analysis provide further evidence of a trilaciclib-mediated antitumor immune response among patients with mTNBC [23, 24]. Patients who received trilaciclib prior to GCb had fewer, but more functional, peripheral T cells and fewer MDSCs after two treatment cycles than patients who received GCb alone. Furthermore, peripheral T-cell numbers, function, and activation were maintained in trilaciclib responders after two treatment cycles but reduced in non‑responders, whereas Treg numbers were maintained and MDSCs were significantly reduced in both groups.

These findings support prior research indicating that, following transient G1 arrest, the proportion of immunosuppressive cells in the tumor microenvironment is decreased and effector T-cell function is enhanced [15]. The benefits of transient T-cell inhibition have also been shown in a drug-regulatable platform, wherein transient inhibition of chimeric antigen receptor expression directed T cells to a memory-like phenotype and restored antitumor functionality in a cell population that had already developed features of exhaustion [33]. CDK4/6 inhibition has previously been shown to enhance T-cell function via the de-repression of the Nuclear Factor of Activated T cell (NFAT) family of proteins. De-repression leads to the activation of downstream genes that regulate T-cell function, resulting in reduced proliferation but increased tumor infiltration and activation of effector T cells [20]. A reduction in the number of MDSCs, which are critical drivers of immune suppression in the tumor microenvironment, suggests that trilaciclib can reduce suppression of the host antitumor immune response to enhance immune-mediated antitumor responses [34].

Data from diagnostic tumor samples collected at baseline showed that genes involved in immune cell activation were upregulated among trilaciclib responders, and there was a trend toward higher TIS scores. In general, although higher TIS scores are not associated with increased OS in breast cancer, they are associated with improved prognosis in those patients with the highest 10% of TIS scores [30]. All KEGG pathways that were upregulated at baseline in trilaciclib responders were pathways that are involved in the generation of an immune response and, except for the graft-versus-host disease pathway, are also necessary for an antitumor response.

A trend for higher T-cell exhaustion at baseline may indicate that patients had a greater existing immune response and, consequently, higher existing T-cell infiltration into the tumor. Exhausted T-cell profiles in the tumor microenvironment [35], or in peripheral blood [36], have previously been associated with better responses. Preclinical studies have shown that, following CDK4/6 inhibition, intratumoral CD8+ T cells display markedly reduced expression of the inhibitory immune receptors PD-1, Tim-3, CTLA-4, and LAG-3—all markers of T-cell exhaustion [37]—potentially enhancing the susceptibility of such tumors to antitumor immune responses. It is possible, therefore, that differential gene expression profiles at baseline, including T-cell exhaustion, may be predictive of response to trilaciclib-containing regimens.

Additional analyses were conducted to compare results by PD-L1 status at baseline. Increased levels of peripheral memory CD8+ T cells and naïve CD8+ T cells were observed after two cycles in trilaciclib responders, regardless of PD-L1 status. However, greater peripheral immune responses and a trend toward an enriched TIS were identified in PD-L1-positive trilaciclib responders at baseline compared with non‑responders. These data support previous research showing that CDK4/6 inhibition promotes the formation of memory CD8+ T cells, which is proposed to occur via upregulation of MXD4 and resultant downregulation of Myc activity during T-cell activation [38]. Furthermore, because an enriched tumor microenvironment suggests better immune cell infiltration, collectively, these data may explain, at least in part, why subgroup analysis of the final OS results from this trial demonstrated larger OS benefit in the PD-L1-positive population [24].

Limitations of this study include the small sample size, particularly in the responder subsets. Moreover, antitumor efficacy outcomes were not the primary study endpoints. The sample size was powered to show superiority among patients who received trilaciclib prior to GCb over those who received GCb alone for at least one primary endpoint (duration of severe neutropenia in cycle 1 or occurrence of severe neutropenia during the treatment period). As such, comparisons of secondary endpoints, including antitumor responses, should be considered exploratory and interpreted with caution. However, the findings of this hypothesis-generating analysis were consistent with, and supportive of, a previous exploratory analysis of the same study [24] and suggest that the improvement in OS observed in patients with mTNBC could be due to increased antitumor immunity, mediated by trilaciclib [24]. Clinical trials are ongoing to explore this further. The phase III PRESERVE 2 trial, investigating trilaciclib or placebo administered prior to GCb in patients with locally advanced unresectable TNBC or mTNBC, has OS as the primary endpoint (NCT04799249). In this study, the impact of trilaciclib on tumor-associated immune responses will be evaluated by comparing immunophenotypic changes between tumor biopsies from patients receiving trilaciclib or placebo. In addition, a phase II mechanism-of-action trial in the neoadjuvant TNBC setting is underway (NCT05112536). The primary objective is to evaluate the immune-based mechanism of action of trilaciclib after a single dose, as measured by changes in the CD8+/Treg ratio in tumor tissue. Pathologic complete response, safety and tolerability, and additional exploratory immune biomarker endpoints will also be assessed.

Overall, these data contribute to a growing body of evidence that transient administration of trilaciclib prior to GCb may enhance antitumor efficacy by both protecting immune cells from chemotherapy-induced damage and modulating the composition and response of immune cell subsets. Data from ongoing clinical studies are critical to confirming the underlying immune mechanisms and to identifying biomarkers that will clearly distinguish between trilaciclib responders and non‑responders.

## Data Availability

All data generated or analyzed during this study are included in this published article.

## Abbreviations

C1D1: Cycle 1, day 1
C3D1: Cycle 3, day 1
CD: Cluster of differentiation
CDK4/6: Cyclin-dependent kinase 4/6
ES-SCLC: Extensive-stage small cell lung cancer
FDR: False discovery rate
FFPE: Formalin fixed, paraffin embedded
FPKM: Fragments per kilobase of exon per million mapped reads
GCb: Gemcitabine plus carboplatin
GSEA: Gene set enrichment analysis
HLA-DR: Human leukocyte antigen–DR isotype
IFN(γ): Interferon (gamma)
KEGG: Kyoto Encyclopedia of Genes and Genomes
MDSC: Myeloid-derived suppressor cell
mTNBC: Metastatic triple-negative breast cancer
NK: Natural killer
NOD: Nucleotide-binding oligomerization domain
OS: Overall survival
PD-L1: Programmed death-ligand 1
RECIST: Response Evaluation Criteria in Solid Tumours
RSEM: RNA-Seq by Expectation Maximization
TIS: Tumor Inflammation Signature
TNBC: Triple-negative breast cancer
Treg: Regulatory T cell
